# Navigation versus experience: providing training in accurate lumbar pedicle screw positioning

**DOI:** 10.1007/s00402-019-03206-7

**Published:** 2019-05-24

**Authors:** L. Leitner, G. Bratschitsch, Patrick Sadoghi, G. Adelsmayr, P. Puchwein, A. Leithner, R. Radl

**Affiliations:** 1 Department of Orthopedics and Trauma, Medical University of Graz, Auenbruggerplatz 5, 8036 Graz, Austria; 2 Department of Radiology, Medical University of Graz, Graz, Austria

**Keywords:** Spinal fusion, Pedicle screw, Learning curve, Training, Navigation

## Abstract

**Purpose:**

Accurate placement of spinal pedicle screws (PS) is mandatory for good primary segmental stabilization allowing consequent osseous fusion, requiring judgmental experience developed during a long training process. Computer navigation offers permanent visual control during screw manipulation and has been shown to significantly lower the risk of pedicle perforation. This study aims to evaluate whether safety, accuracy, and judgmental skills in screw placement, comparable to an experienced surgeon, can be developed during training using computer navigation.

**Methods:**

Lumbosacral PS were placed in 18 patients in a prospective setting, in one segment side with conventional fluoroscopy by a senior spine-surgeon, and computer navigated on the other side by a trainee without prior experience in the technique. At the beginning and at the end of the study, PS were placed freehand in solid foam models by the trainee. PS placement time, intraoperative placement revisions, PS placement accuracy on postoperative CT scans, and postoperative complications were assessed.

**Results:**

Significant improvement of trainee’s PS placement accuracy (Sclafani score 8.2–8.83; *p* = 0.006) and time (13.3–6.8 min per screw; *p* = 0.005) to a similar level as the experienced surgeon state (5.2–4.1 min per screw; *p* = 0.39) was explored; similar improvement was explored in the foam models. The number of intraoperative placement revisions kept on a low level for surgeon (3.3–0.0%) and trainee (5.1–2.6%) during the whole study, no postoperative complications occurred.

**Conclusion:**

Navigated PS insertion allows safe teaching from the early beginning of surgical training, due to steady intraoperative control on PS placement. Adequacy of PS placement is similar to screws placed by an experienced surgeon. Progress in judgmental skills in screw placement can be gained rapidly by the trainee, which can also be transferred to non-computer navigated PS placement.

## Introduction

Pedicle screw (PS) placement, which is considered complex and technically demanding, is a widely used technique for surgical correction of deformities, degeneration, infection, malignancy, and trauma of the spine. Adequate PS placement presents an important predictor for postoperative outcome [1] and misplacement rates up to 30% with non-navigated screw insertion have been described [2]. A range of navigation systems was made available, allowing image-guided screw insertion, intended to reduce the risk of pedicle perforation [3] and increase accuracy in placement of lumbar PS [4, 5]. However, drawbacks of these techniques have been considered: investment costs, lack of availability and training opportunities, and increased time-consumption due to interruption of the work flow have been pointed out [6]. There are also prospective studies and meta-analysis, which failed to show significant improvement of accuracy or clinical advantage in reduction of neurological symptoms of computer-navigated PS placement techniques [7–9]. Still computer-navigated PS placement seems recommendable in especially complicated spine surgeries, due to significant deformity, revision surgeries, or complex trauma, to ensure accuracy [3, 10, 11].

Published learning curve analysis revealed, that computer-navigated PS placement offers high accuracy of lumbar PS placement from the beginning [12] and radiation exposure to the surgeon is reduced [13]. Based on these findings computer navigation may be favourable for novice operators to obtain initial experience in spine surgery, offering real-time control and allowing broad adjustment of arrangement during PS placement without increasing radiation exposure. However, the exact feasibility of computer navigation in training concerning safety, accuracy, and development of judgmental skills is still elusive.

The aim of our study was therefore to evaluate the feasibility of computer navigation as training method for PS placement, concerning safety, accuracy, and development of judgmental skills. The hypothesis was that the observed parameters would significantly improve for the trainee using navigation for PS placement.

## Methods

### Study population and data

This study was approved by the local Institutional Ethical Review Board (reference number: 27-444 ex 14/15). All experiments were performed in accordance with relevant guidelines and regulations; informed consent was obtained from all participants.

All surgical procedures were performed by the same orthopedic spine surgeons (RR, ‘experienced’ senior surgeon with long-standing expertise in spinal instrumentation; LL, ‘trainee’ resident with no prior surgical expertise in spine surgery). Inclusion criteria were indication for primary posterior fusion of at least one lumbar and/or the lumbosacral segment. Exclusion criteria were revision surgeries and fusion of thoracic segments and/or iliosacral fusion. A median of three motion segments (range 1–4) was instrumented per patient. Patients’ demographic characteristics (age, sex, BMI) were retrieved from our hospital database system.

### Operations agenda

Eighteen patients under general anesthesia were positioned prone on a radiolucent carbon-operating table. Open dorsal spinal instrumentation was performed on one randomly selected side (9 left, 9 right) by the experienced surgeon with conventional fluoroscopy using a C-arm (Siremobil Compact, Siemens, Germany). Afterwards, computer navigation (O-arm Technology, Medtronic, MN, USA) was conducted on the other side by the spine surgery trainee, performing an initial, and a closing O-arm scan after screw placement. In case that the closing scan revealed inadequate screw placement, the experienced surgeon performed replacement using C-arm fluoroscopy-guided technique. Time measurement for each surgeon was started at the beginning of entry point preparation with a bone awl. PS placement time per screw and screw revisions during surgery were recorded.

### PS accuracy

Accuracy of PS placement of each surgeon on closing O-arm scan was analyzed according to earlier published classifications. In short, Gertzbein and Robbins (G&R) classification for pedicle containment of lumbar and sacral PS [2] describes cortical pedicle breaches by the extent of extracortical screw violation (grade 0, no evidence of violation; all higher grades, where cortical breach distance is measured from the medial border of the pedicle were considered as evidence of violation). Accuracy score by Sclafani et al. [14] awards grades for screw length; axial and sagittal trajectory; medial, sagittal and lateral containment (score: min–max, 0–10), and was documented for lumbar PS. Scores were documented and rechecked by an independent, blinded radiologist (GA). By inserting non-navigated PS in solid foam lumbar models (Sawbones, Vashon Island, WA, USA) using freehand technique, the skills of the unexperienced trainee were evaluated for time per screw and accuracy in a post-interventional O-arm scan prior to the interventional study. The same procedure was performed after the interventional study, results concerning accuracy and procedural time were compared.

### Statistical methods

Sample size calculation using two-sided test of difference between two mean values (*α* = 0.05, power (1 − *β*) = 0.80) for detection of a mean difference in screw accuracy score of 0.8 with a standard deviation of 1.5, according to earlier published cadaveric learning curves for O-arm navigation [14] was performed prior to the study.

Based on a calculated sample size of 26 lumbar segments, to allow comparison between first half and second half PS inserted, minimum of 52 lumbar segments had to be included. 18 patients (F: 7, M: 11; 53 lumbar segments, 16 sacral segments) undergoing open dorsal instrumentation for spinal degeneration were included in this prospective, controlled study between January 2016 and December 2016. Patients were divided into two subgroups containing initial 9 patients (31 lumbar segments, 8 sacral segments) and final 9 patients (22 lumbar segments, 8 sacral segments) for comparison of time per screw, screw placement accuracy over the course of time, and between the two surgeons. IBM SPSS Statistics 20 (IBM, Armonk, NY, USA) was used for data analysis. Statistical analysis was performed using Chi-squared test for comparison of categorical parameters, *t* test for comparison of continuous normally distributed parameters and Spearman’s correlation coefficient for calculation of correlations. A two-sided *p* value < 0.05 was considered to be statistically significant.

## Results

**Fig. 1:**
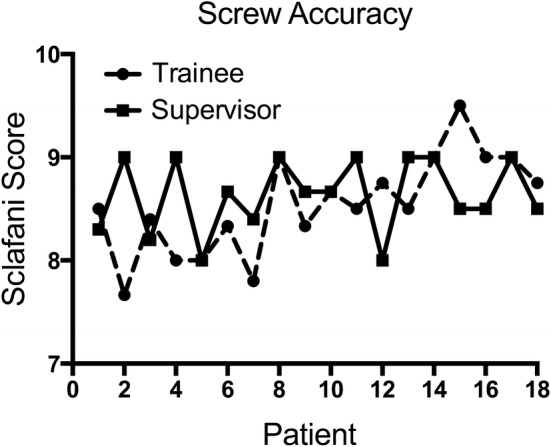
Curves showing mean grading of accuracy according to Sclafani et al. [14] of sacral and lumbar PS of the patients in the course of the study for supervisor (filled squares) and trainee surgeon (filled circles)

**Fig. 2:**
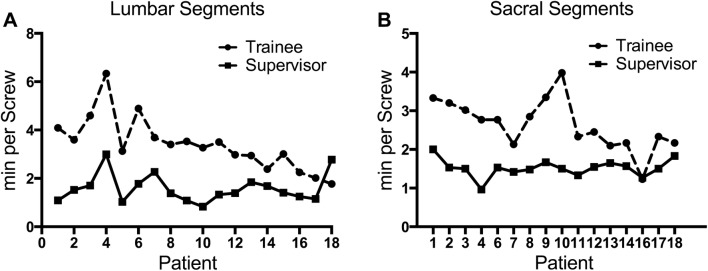
Curves showing mean time needed for **a** lumbar and **b** sacral PS of the patients in the course of the study for supervisor (filled squares) and trainee surgeon (filled circles)

**Fig. 3:**
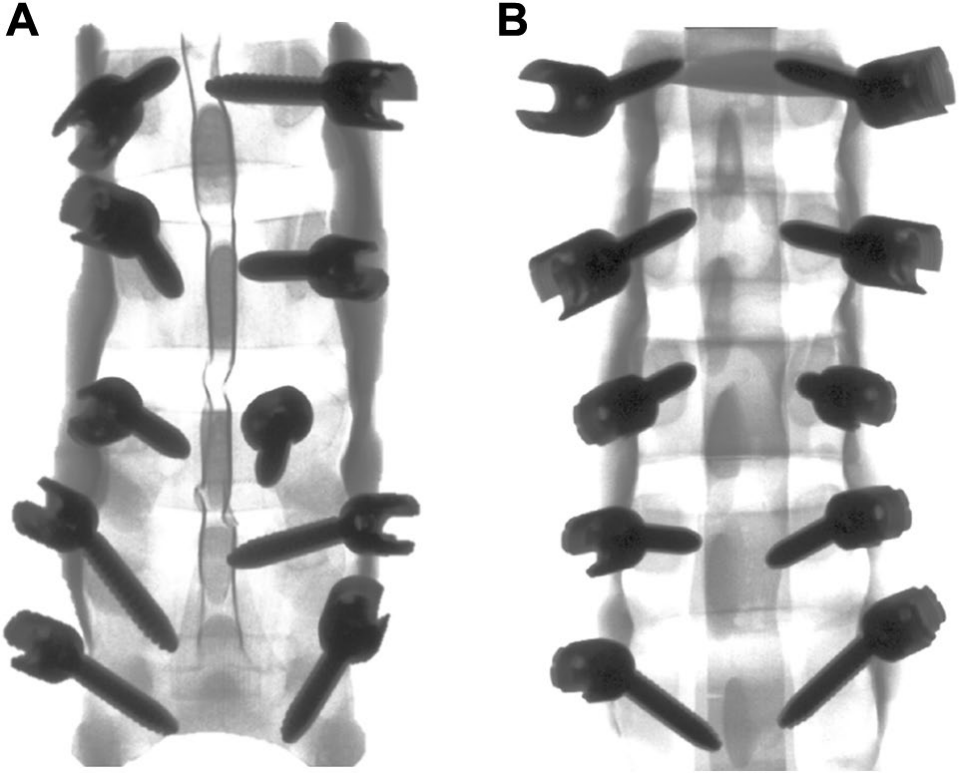
PS were inserted freehand in solid foam lumbar models by the trainee before inclusion of the first patient when the trainee had no experience in PS placement (**a**) and at the end of the study (**b**); a.p. view

A total of 138 screws were placed in 53 lumbar and 16 sacral segments of 18 patients (age 67.1 ± 15.1 years; range 42–85 years). No intra- or postoperative complications or instance of postoperative neurologic deficit or vascular injury were observed within a follow-up of at least 4 months. One patient had to be revised for implant dislocation mainly caused by poor bone quality which occurred 4 months after operation. A significant improvement of screw placement accuracy score according to Sclafani et al. [14] could be explored for the trainee between the initial and the final nine patients (8.2, 8.83; *p* = 0.006; Table 1; Fig. 1). Although, by trend, the trainee improved cortical pedicle violation rate, reaching similar values to the senior surgeon during the study, no significant differences were found in the percentage of PS totally contained within the pedicle according to Gertzbein et al. [2] and screw placement accuracy score between the two surgeons in the two subgroups (Table 1). A significant improvement of PS insertion time was explored for the trainee for lumbar PS placement between the initial and the final half of patients (13.3 min; 6.8 min; *p* = 0.005), whilst the placement time for the senior surgeon remained on a steady state (5.2 min; 4.1 min; *p* = 0.39) (Table 1; Fig. 2). Still, a significant difference in PS placement time between the two surgeons in initial (*p* = 0.001) and final subgroup (*p* = 0.049) remained (Table 1). A similar effect could be explored for sacral screws, even though the trainee’s improvement in placement time was not significant (Table 1). Although time per screw curves between surgeons seemed to exhibit a similar course (Fig. 2), no significant correlation was found between the two surgeons screw placement curves (*r* = 0.17; *p* = 0.5). Rate of PS placed by the trainee, that needed to be revised after initial placement, improved from 5.13 to 3.33% between initial and final nine patients (Table 1). Freehand PS placement in solid foam lumbar model at the end of the study significantly improved concerning cortical pedicle violation rate (1, 0.3; *p* = 0.001) screw placement accuracy score (7.0, 8.2; *p* = 0.003) with similar placement time compared to the performance at the beginning of the study (Table 2; Fig. 3).

**Table 1:** Differences in screw positioning measurements between the initial (*n* = 9) and final (*n* = 9) patient cohort with comparisons between supervisor and trainee surgeon

Patients pedicle screws	Trainee	Supervisor	Significance
Cortical pedicle violation (%) (all segments; *n* = 69)			
Initial group	0.34	0.37	*0.814*
Final group	0.26	0.26	*1.000*
Significance (*p*)	*0.335*	*0.458*	
Accuracy score (1–10) (lumbar segments; *n* = 53)			
Initial group	8.20	8.47	*0.220*
Final group	8.83	8.61	*0.350*
Significance	***0.006***	*0.539*	
Time per screw (min) (lumbar segments; *n* = 53)			
Initial group	13.29	5.25	***0.001***
Final group	6.78	4.06	***0.049***
Significance	***0.005***	*0.387*	
Time per screw (min) (sacral segments; *n* = 16)			
Initial group	2.93	1.51	** < ** ***0.001***
Final group	2.35	1.53	***0.010***
Significance	*0.076*	*0.918*	
Revision rate (%) (all segments; *n* = 69)			
Initial group	5.13	2.56	
Final group	3.33	0.00	

Significant *p*-values < 0.5 are kept in bold and italics

Cortical pedicle violation, according to Gertzbein et al. [2]; Accuracy score, according to Sclafani et al. [14]

**Table 2.** Differences in PS positioning between solid foam lumbar models carried out by the trainee surgeon at the beginning of the study and at study ending

Foam lumbar segments (*n* = 10)	Trainee	Supervisor
Cortical pedicle violation (%) (all segments; *n* = 10)		
First model (pre)	1.00	n.m.
Second model (post)	0.30	n.m.
Significance	***0.001***	n.m.
Accuracy score (1–10) (all segments; *n* = 10)		
First model (pre)	7.00	n.m.
Second model (post)	8.2	n.m.
Significance	***0.003***	n.m.
Time per screw (min) (all segments; *n* = 10)		
First model (pre)	4.4	n.m.
Second model (post)	4.7	n.m.

Significant *p*-values < 0.5 are kept in bold and italics

Cortical pedicle violation, according to Gertzbein et al. [2]; accuracy score, according to Sclafani et al. [14]

## Discussion

After initial euphoria regarding computer navigation techniques for PS placement, chastening results concerning superiority of navigated techniques, regarding accuracy of pedicle screw placement, clinical outcome and economic aspects, have been explored and published by several authors [7, 9, 15]. The earlier explored low correlation between accuracy of screw placement, which will most evidently be improved with navigation, and clinical outcome in PS placement [16] might provide an explanation for the difficulty to effectively show a possible superiority. Some authors proposed that patient selection might play an important role for advantageous utilization of navigated PS insertion. Due to better overview on the current anatomy, navigation systems may be particularly required in complex cases as deformity, trauma, and revision surgeries [3, 10, 11].

In this study, we assumed that it might also depend on the type of surgeon, who is ‘selected’ to realize highest benefit from computer-navigated PS insertion. This assumption was based on earlier findings, that experienced surgeons achieve high PS accuracy using non-navigated PS insertion technique (thoracic spine; no pedicle breach in 97.5%) [17], whilst on the other hand for novice spine surgeons a learning interval with a clearly higher amount of inadequate PS placement has been described (cadaver, thoracic spine; adequate PS in 71.0%) [18]. This could vice versa implicate, that the highest benefit can be achieved by an unexperienced surgeon.

Therefore, an unexperienced surgeon was selected for PS placement using O-arm computer-navigated technology, to measure his improvement in PS placement accuracy and time. Permanent real-time control on the trainee during the screw positioning process provided by O-arm technology revealed in a low revision rate for failed screw placement, decreasing from an initially low level of 5.1% of PS to a revision rate of 3.3% at the end of the study. These results suggest that navigated PS insertion already provides a high safety for the patient from the early beginning of spine surgery training.

A highly adequate PS insertion result was reached by the trainee from the very first patient, with a steady increase, in the end appearing tangentially better, result compared to the experienced surgeon (Fig. 1). In this clinical setting, the trainee’s PS placement adequacy from the very beginning was comparable to earlier published reports on PS adequacy in patients in the literature [3, 14]. Significant improvement was also explored concerning enhancement in PS insertion time as shown by our data (Fig. 2).

Although this study did not focus on radiation dose to surgeon or patient, a reduction seems plausible when O-arm computer navigation technology is used for spine surgery training. We could show that adjustments and replacements, which—according to our experience—are performed by unexperienced surgeons to a higher extent, can be markedly reduced and withal, if necessary, do not cause additional radiation when O-arm technology is used.

Experience is based on judgmental skills according instrument handling, entry point direction, changes in resistance during hammering, and drilling as earlier experiments attempted to elaborate [18]. Still, it could not be closed out that permanent reliance on visual feedback provided by the computer navigation system counteracts the gain in judgmental skills in PS placement. Especially freehand placement of PS requires substantial judgmental skills and good anatomic knowledge [18]. Using the lumbar model at the beginning and end of the study for freehand PS placement by the trainee, mainly concerning on anatomical landmarks and tactile feedback during screw insertion, was our attempt to evaluate whether judgmental skills are gained using navigation technique, and whether they can be transferred to conventional screw placement. Our results from these experiments suggest that a substantial gain in judgmental skills could be elaborated with this technique, resulting in significantly improved PS positioning (Table 2; Fig. 3).

### Limitations

Although PS placement and insertion were performed by the trainee himself, the senior physician provided advice on PS’ placement, if necessary (i.e., handling of instruments). This effect cannot be determined appropriately, and could add a fractional explanation why screw placement accuracy did not show significant differences between the two surgeons at any time. On the other hand, this limitation has no influence on the question whether O-arm computer navigation is a safe teaching method. Especially entry point identification, which can be demanding at the beginning of spine surgery training, was safe and easy to achieve with O-arm technology without demand of supervision or correction.

Furthermore, it should be mentioned, that adequate implementation of O-arm technique is an absolute requirement for reproducibility of our results. Previous studies identified a multifold of possible pitfalls (e.g., unintentional modifications of the tracking device, poor image quality in obese) leading to drawbacks in use of navigation technologies [12]. In our setting, the senior surgeon was well educated in the use of O-arm technology due to regular use.

## Conclusion

The use of O-arm technology in spine surgery training seems to provide a rapid gain in judgmental skills concerning PS placement, which might also be transferred to freehand PS placement. Adequacy of PS placement by a trainee using navigation was similar to screws placed by an experienced surgeon. The supervisor keeps permanent, real-time visual control on the PS placement process, offering a well-balanced combination of surgical training and patient safety. As conclusion we recommend that navigation technology as supportive technique should be used in spine surgery training whenever it is available, taking into consideration that exact anatomic skills are still required.
